# Making Room for Every Voice: Reimagining Person‐Centred Care in the Neurosciences

**DOI:** 10.1111/hex.70350

**Published:** 2025-07-29

**Authors:** Miguel Toribio‐Mateas, Gareth Noble

**Affiliations:** ^1^ School of Psychology, Psychology Tower Building Cardiff University Cardiff UK; ^2^ Medical School Swansea University Swansea UK

**Keywords:** co‐creation, community engagement, equity in healthcare, lived experience, neurosciences, person‐centred care

## Abstract

**Background:**

This Special Issue of *Health Expectations* brings together 38 articles that exemplify the growing commitment to person‐centred care in the neurosciences. Moving beyond a historically brain‐centric model, these contributions reflect a more human discipline that values lived experience alongside clinical expertise.

**Methods/Results:**

Spanning a wide range of neurological conditions, life stages and care settings, the papers explore interconnected themes including co‐creation, identity, equity, communication, service redesign, emotional well‐being, innovation and community engagement. The authors in this collection demonstrate that care becomes not only more effective but also more ethical when people are recognised as active partners rather than passive recipients. From co‐designed tools and culturally responsive resources to narrative inquiry and community‐led research, these works reveal the transformative power of relational approaches that reorient systems, promote autonomy and respond to what truly matters to those living with neurological conditions.

**Conclusions:**

Together, these articles challenge longstanding hierarchies and power dynamics, advocating for care that is grounded in trust, reciprocity and compassion. They are more than a snapshot of current practice; they are a call to action, a provocation to imagine what neurological care could become. At its heart, this Special Issue invites researchers, clinicians and communities to co‐create a neuroscience that is more advanced and more attuned to the lives it seeks to serve.

**Patient or Public Contribution:**

This Special Issue was developed with a focus on highlighting the voices, experiences and expertise of people living with neurological conditions, their families and communities. Many of the 38 articles featured were co‐produced or co‐authored with individuals with lived experience, and we, as Guest Editors, have intentionally curated the issue to centre these perspectives. While patients or members of the public were not directly involved in the writing of this Editorial, their contributions are reflected throughout the Special Issue in the form of participatory research, co‐created resources and narrative accounts. The Editorial itself is informed by these works and seeks to honour their insights by amplifying their relevance and impact within the broader neuroscientific community.

Over recent years, the delivery and organisational structures of healthcare have been re‐designed to encompass a patient‐centred and value‐based approach that ensures that patients are at the heart of their healthcare. Neuroscience is defined as a discipline that informs neurological patient care, across a range of conditions, that reflects individual patient needs, supports patient engagement, embeds shared decision‐making and recognises the importance of acknowledging patients' own lived experiences. The integration of a patient‐centred approach into the research exploration of neuroscience and its application into neurological care supports patient partnership and consideration of the whole person and their quality of life.

The neurosciences are being transformed into a more human discipline, one that values and integrates people's lived experiences into clinical practice, research and innovation. This shift is far from theoretical; it is visible in how we ask questions, design studies, deliver care, and engage with people and communities. For too long, the focus in neurology has been on the brain in isolation. Now we are listening more intently to the person behind the brain scan. This Special Issue of *Health Expectations* is a celebration of that shift, a collection of articles that evidences the extent of the commitment of researchers and clinicians to person‐centred care in neurosciences and neurological care. The overarching theme of this collection of articles is how research and clinical practice have demonstrated the value of patient‐centred care in informing patient outcomes. It brings together a rich array of work from researchers, practitioners, people living with neurological conditions, and their allies, each contributing to a more relational, compassionate and participatory approach to care. The 38 articles span a wide range of conditions, age groups and social contexts, but all converge on a shared commitment: to listen to, involve and respond to what truly matters to people living with neurological conditions, their families and their communities.

The work presented in this issue explores that commitment through a number of interwoven threads. Each paper is a rich source of compelling insights into what it takes to make care genuinely person‐centred, reminding us that progress often begins with simply asking, ‘*What matters to you?’*

## 1 Co‐Creation, Participation and Agency

Across this Special Issue, one idea stands out with particular clarity: care becomes more effective, and indeed more human, when the people it is designed for are treated not as subjects or defined by diagnostic labels, but as partners. Many of the authors here embrace co‐creation not as a methodological choice, but as an ethical stance.

Kuroda et al. [1] begin with a simple but profound shift: asking people with mild cognitive impairment what they want to know, and building a handbook around their questions. In doing so, they show how participation reorients both the content of care and its purpose. Veerhuis et al. [2] extend this principle across cultural boundaries, adapting a dementia and driving decision aid through ongoing dialogue with people living with dementia and their families, resulting in a tool that is both contextually grounded and emotionally respectful.

This relational design theme continues in Hohl et al. [3]'s work, where the creation of an information resource on disorders of consciousness becomes a model of co‐production that is scientifically robust, emotionally attuned and clinically relevant. In another contribution, Dosso et al. [4] provide a striking example of inclusive innovation: their collaboration with young people to develop socially responsive robotics for paediatric anxiety fuses clinical insight with empathy, creating technology that goes beyond function and into feeling. In parallel, Geerts et al. [5] work closely with people living with small fibre neuropathy to craft a patient satisfaction measure that genuinely reflects the complexity, frustration and nuance of diagnostic experiences and informs healthcare services.

These are not isolated efforts. They are part of a broader shift throughout this collection towards care that is not done to people, but built with them, thereby challenging the traditional dynamics of power in clinical research. Shrubsole et al. [6] bring this ethos into speech and language therapy, developing quality indicators for aphasia services that emerge from the priorities of those most affected. Tuvemo Johnson et al.'s [7] fall prevention programme succeeds because it speaks in the language and logic of its users.

The importance of integrated, person‐centred care across neurodegenerative conditions is powerfully highlighted by Bartolomeu Pires et al. [8], whose systematic review synthesises current best practice in person‐centred integrated care for people living with Parkinson's disease, Huntington's disease and Multiple Sclerosis (MS). Their work distils the key ingredients of effective, collaborative models, emphasising the value of coordination, communication and genuine partnership across the care pathway.

Wills et al. [9] (Olivia) show how lifestyle support in MS becomes more effective, and more sustainable, when care teams act as facilitators rather than gatekeepers. And in one of the clearest expressions of this theme, Kennedy et al. [10] demonstrate how community‐driven research not only surfaces new questions, but reshapes what we think research is for and how it can translate into healthcare practice.

## 2 Identity, Meaning and Emotional Well‐Being

Neurological diagnoses often shake the foundations of identity, prompting a reweaving of one's sense of self in the face of uncertainty, change and loss. This theme resonates powerfully across several contributions in this issue. Faccio et al. [11] put forward a poignant and nuanced account of how people navigate the aftermath of acquired brain injury, reconstructing identity in ways that are deeply personal and emotionally complex. In a different, but equally affecting context, Howard et al. [12] explore the psychological landscape of living with the genetic risk of motor neuron disease, where *not knowing* becomes a constant companion and shapes daily life in enduring ways.

The challenges of identity and adaptation are further explored by Aspö et al. [13, 14] in two thoughtful papers: one examines how people experience the diagnostic process in memory clinics, while the other traces the evolving sense of self and place following a diagnosis of young‐onset Alzheimer's disease. These studies speak to the emotional terrain that often remains hidden beneath clinical assessments.

Through a more narrative lens, Hussain‐Ali et al. [15] provide an evocative reflection on young‐onset Parkinson's disease, drawing attention to the gendered dimensions of illness and reminding us whose experiences have too often been marginalised. That same depth of emotional insight runs through the work of Smith et al. [16]. Their photovoice study lends form and visibility to the non‐motor symptoms of Parkinson's disease, while their earlier work on vestibular disorders captures the subtle disruptions these conditions create in one's emotional and relational world.

Elsewhere, Luckhaus et al. [17] explore self‐care in Parkinson's disease not as an abstract goal but as a lived, fluctuating negotiation between autonomy, identity and vulnerability. And in a valuable article focused on children, Lin et al. [18] focus on the voices of young people with tethered spinal cord syndrome, showing us how neurological conditions affect not just the body, but one's sense of belonging and participation in everyday life.

## 3 Navigating Access, Equity and Structural Barriers

Access to care is never just about availability; it is entangled with geography, language, culture and historical marginalisation contributing to the health inequalities communities may encounter when accessing and/or receiving healthcare services. Many of the articles in this issue confront these realities head‐on, revealing the ways in which systems tend to fall short and how they might begin to do better.

O'Shea et al. [19] expose the structural inequities faced by people with Parkinson's disease in Ireland, where patchy service provision and inconsistent support can leave individuals feeling isolated and underserved. In response to a different kind of gap, Ali et al. [20] demonstrate the transformative potential of language. Their work on multilingual dementia awareness materials shows how co‐production can bridge cultural divides and bring relevance to communities that are often overlooked in mainstream healthcare communication.

Some inequities, however, are more deeply rooted and more urgent. Wills et al. [21] (Elaine) provide a powerful and unflinching account of Indigenous women living with traumatic brain injury as a result of family violence. Their article stands out not just for its subject matter, but for its commitment to making space for voices that health systems have historically silenced.

The aftershocks of the Covid‐19 pandemic are also present in this theme. Sakel et al. [22] examine how people living with spasticity experienced service disruptions. Their paper is a stark reminder that recovery from the pandemic has been anything but equal. These disruptions reveal how fragile support systems can be, particularly for those with complex and ongoing needs.

In a similar vein, Wills et al. [23] (Olivia) bring a socioecological perspective to the experience of being newly diagnosed with MS. Their study highlights how self‐management is shaped not just by personal motivation but by relationships, environments and systems that may either support or obstruct autonomy. And finally, Dilger et al. [24] add an important voice to this conversation by focusing on mobility and participation. Through this lens, they reveal the barriers that people with gait impairments must navigate every day, often invisibly, within communities and care structures that are not yet built for them.

## 4 Communication, Shared Decision‐Making and Advocacy

The heart of person‐centred care lies not only in what we do, but in how we communicate, how we talk with people, how we listen, and how we make decisions together. Several authors in this issue bring depth to these seemingly simple, but frequently overlooked, interactions.

Damman et al. [25] explore how outcome measures in MS consultations can act as tools for shared decision‐making through meaningful engagement, but, importantly, they highlight that this only happens when they are used as a springboard for genuine dialogue rather than bureaucratic box‐ticking. In a similarly thoughtful contribution, Papadimitriou et al. [26] share a relationship‐centred decision‐making model that reimagines rehabilitation not as a service delivered to patients, but as a co‐authored process rooted in mutual understanding, respect and trust.

In a fascinating intersection of overlapping themes, Ownsworth et al. [27] reveal the power of self‐advocacy following acquired brain injury, showing how individuals can reclaim agency and challenge the assumptions built into care systems. And, bringing carers' voices into focus, Shawaqfeh et al. [28] put forward a grounded view of the real‐world complexity involved in decisions around anticholinergic medications in dementia care. Their work reminds us that advocacy unfolds in the emotionally charged moments of everyday life rather than in formal meetings.

Clinical routines, too, are not immune to scrutiny. Aspö et al. [13] show how even standard memory clinic assessments can either strengthen trust or sow confusion and doubt, depending on how they are framed and delivered. And in another example of thoughtful design, Veerhuis et al. (2024) return with a co‐created decision aid that supports, rather than substitutes, human dialogue. These studies collectively emphasise that communication in healthcare is a lot more than ‘a soft skill’; it's a clinical intervention in its own right.

## 5 Service Redesign, Professional Practices and System Gaps

If we are to realise the promise of person‐centred care, we must also be willing to reimagine the healthcare systems that shape it. Several authors in this issue turn a critical eye to the institutional structures and professional cultures we often take for granted.

Bright et al. [29] bring visibility to the invisible labour of stroke clinicians striving to prioritise psychosocial needs in services built around physical outcomes. In doing so, the authors expose a tension that many practitioners will recognise: the push to deliver holistic care in systems that reward speed and standardisation. Anemaat et al. [30] give voice to speech pathologists working in aphasia care, whose reflections reveal a quiet resilience and a hunger for change.

Meanwhile, Kaş et al. [31] use metaphor analysis to illuminate the emotional landscapes of families caring for loved ones at risk of aspiration. Through their lens, we see how fear and uncertainty are carried not just in words, but in images, silences and half‐spoken worries.

Some authors highlight the small‐scale interventions that make a big difference. Chaudhry et al. [32] show how a telephone helpline for stroke survivors can serve as a vital bridge, connecting people to resources, reassurance and a sense of continuity at a time when care often feels fragmented. And Nguyen et al. [33] transport us into the future of neurotherapeutics, inviting us to reflect not only on the promise of these cutting‐edge interventions, but also on their ethical complexity. This study is a reminder that innovation must be grounded in dialogue with those it aims to help.

## 6 Psychosocial Support and Recovery in Context

Neurological care that neglects the emotional and social dimensions of illness will always fall short. This theme emerges powerfully in several papers that challenge us to see beyond the clinical diagnosis.

Van Rijn et al. [34] examine the experiences of people with severe mental illness during the Covid‐19 pandemic, highlighting the double burden of isolation and systemic neglect. Their findings are sobering, but they also cast light on pathways to more resilient, community‐rooted models of support.

This is echoed in Smith et al.'s [35] work on vestibular disorders, which underscores how even relatively ‘invisible’ conditions can disrupt a person's emotional and relational world, while Addington et al. [36] shed light on a rarely discussed issue: pelvic floor dysfunction in women with MS. This perspective broadens the scope of what counts as relevant in neurological care, validating symptoms that are too often minimised or ignored.

Returning to the theme of embodied realities of mobility impairment, Dilger et al. [24] explore how gait difficulties shape not only participation but also identity and self‐perception. And echoing another core theme in this issue, Howard et al.'s [12] exploration of genetic risk brings us back to the emotional labour of uncertainty, the ongoing task of holding possible futures in tension with present‐day life. Together, these contributions insist that care must address the whole person, not just the condition.

## 7 Innovation, Education and the Promise of Technology

Innovation in the neurosciences can feel exciting, but it risks being dislocated from the lives of those it affects unless grounded in real human needs. Several authors in this issue show us how to bridge that gap.

Nguyen et al. [33] explore the hopes, concerns and priorities of families and professionals engaging with advanced therapeutics for rare neurological diseases. Their findings serve as a reminder that innovation is never neutral and must be shaped by the values of those who will live with its outcomes.

Roeser et al. [37] highlight the work of the F.A.S.T. Council, demonstrating how lived experience can be effectively integrated into advocacy and policy for Alzheimer's disease. Their approach reframes education not as the transfer of knowledge from experts to laypeople, but as a process of collective meaning‐making.

And in a return to earlier themes, Dosso et al.'s (2024) robotics project reminds us that technology, when designed with rather than for its users, can become a vehicle for connection, not alienation. These papers call for a vision of innovation that is ethical, inclusive and grounded in everyday realities of patients' lived experiences.

## 8 Community Engagement and Collective Learning

Some of the most transformative insights in this Special Issue come not from formal institutions, but from communities, from the people who live with neurological conditions, support each other, and build new forms of care from the ground up.

Eisenhut et al. [38] explore therapeutic volunteering, revealing how structured opportunities for contribution can support both well‐being and social connection for people with long‐term neurological conditions. Wills et al. [23] (Olivia), in their second study, show how MS care teams can act as relational bridges, helping translate medical advice into everyday action in ways that feel sustainable and meaningful, in a way that could empower patients.

The power of co‐produced research to shape not just findings, but futures is illustrated by Kennedy et al. (2025) and Wills et al. (2025) (Elaine). Their focus serves as a reminder that when communities guide the questions, the answers become more relevant and more just. Hohl et al.'s (2025) educational materials for carers of people with disorders of consciousness further exemplify this ethos: collaborative, informative and rooted in compassion.

And in a fitting closing note, Chaudhry et al. [32] remind us that, sometimes, care begins with the simplest of gestures: a listening ear at the other end of the line. In other words, a human connection is not an ‘extra’; it is the essence of support.

## 9 Conclusion

This Special Issue exemplifies the breadth and depth of person‐centred care in neurosciences. Across all articles, there is a shared insistence that care must be co‐created, equity‐focused and responsive to people's lives in all their complexity. Whether in the design of a decision aid, the architecture of a care pathway or the nuance of a clinical encounter, the message is clear: the future of neurological care lies not in ever‐greater technological sophistication alone, but in the consistent and compassionate application of person‐centred principles.

We hope these contributions inspire further transformation across disciplines, systems and, most importantly, relationships of care.

As Guest Editors, it's been a privilege to curate this Special Issue alongside such thoughtful, committed contributors. We hope it serves as a catalyst for further dialogue, collaboration and change.

Miguel Toribio‐Mateas, Honorary Research Fellow, School of Psychology, Cardiff University

Gareth Noble, Medical School, Swansea University.

## Author Contributions

Miguel Toribio‐Mateas was responsible for conceptualisation, formal analysis, and writing the original draft. Gareth Noble contributed to review and editing of the manuscript. Both authors contributed to the interpretation and discussion of the themes and approved the submitted manuscript.

## Conflicts of Interest

The authors declare no conflicts of interest.

## Data Availability

Data sharing is not applicable to this article, as no new data were created or analyzed in this study.
